# The statistical software revolution in pharmaceutical development: challenges and opportunities in open source

**DOI:** 10.1016/j.drudis.2026.104613

**Published:** 2026-03

**Authors:** Daniel Sabanés Bové, Heidi Seibold, Anne-Laure Boulesteix, Juliane Manitz, Alessandro Gasparini, Burak K. Günhan, Oliver Boix, Armin Schüler, Sven Fillinger, Sven Nahnsen, Anna E. Jacob, Thomas Jaki

**Affiliations:** 1 RCONIS/inferential.biostatistics GmbH, Friedrichstrasse 12, 4055 Basel, Switzerland; 2 Digital Research Academy, Bayerstrasse 77c, 80335 Munich, Germany; 3 Institute for Medical Information Processing, Biometry and Epidemiology, Faculty of Medicine, LMU Munich, 81377 Munich, Germany; 4 Munich Center for Machine Learning, Ludwig-Maximilians-Universität München, Institut für Informatik, Oettingenstraße 67, 80538 München, Germany; 5 R Validation Hub, c/o R Consortium, 101 Spear Street, Suite 550, San Francisco, CA 94105, USA; 6 Department of Medical Epidemiology and Biostatistics, Karolinska Institutet, PO Box 281, 17177 Stockholm, Sweden; 7 Red Door Analytics AB, Valfrid Palmgrens gata 4, 11369 Stockholm, Sweden; 8 Merck Healthcare KGaA, Frankfurter Strasse 250, 64293 Darmstadt, Germany; 9 Bayer AG, Aprather Weg 18a, 42113 Wuppertal, Germany; 10 Biostatistics and Special Pharmacokinetics Unit, Federal Institute for Drugs and Medical Devices (BfArM), 53175 Bonn, Germany; 11 Quantitative Biology Center (QBiC), University of Tübingen, 72076 Tübingen, Germany; 12 Biomedical Data Science, Department of Computer Science, University of Tübingen 72076 Tübingen, Germany; 13 National Food Institute, Technical University of Denmark, Anker Engelunds Vej 1, 2800 Lyngby, Denmark; 14 Faculty of Informatics and Data Science, University of Regensburg, Bajuwarenstrasse 4, 93053 Regensburg, Germany; 15 MRC Biostatistics Unit, University of Cambridge, East Forvie Building, Forvie Site, Robinson Way, Cambridge CB2 0SR, UK

**Keywords:** software, open source, statistics, clinical trials, pharmaceutical industry, academia

## Abstract

Open-source statistical software development is increasingly the preferred solution for leveraging new statistical methods in the pharmaceutical industry. However, with a long history of relying on licensed analysis software, there are philosophical and organizational barriers to overcome. In particular, the sustainability, reliability, usability, and feasibility of maintaining open-source statistical software long-term must be ensured. Here, we describe the open-source revolution that is emerging in the pharmaceutical industry and how it facilitates greater scaling of innovative analytical methods in statistics. We discuss challenges to open-source software adoption and propose mitigation strategies. Furthermore, we illustrate the potential for open-source software development with examples of successful projects, which highlight the roles of cross-company collaboration, career paths, education, and community building.

## Introduction

The pharmaceutical industry has been described by many as having serious challenges with productivity, efficiency, and innovation.^(p1)^^,^^(p2)^^,^^(p3)^ Productivity, as assessed by the number of new drug approvals per year, has remained relatively flat, while the research and development (R&D) spend per approval has increased dramatically.^(p4)^ Various reasons have been posited, including changes in portfolio composition (e.g., entering new high-risk therapeutic areas with lower success rates), greater complexity in some therapeutic areas because of greater competition, advances in technology leading to a wider array of potential targets, and the high degree of regulation governing the industry. A generation of leaders has responded to these challenges by investing in myriad new technologies. Examples include high-throughput screening (HTS), whole-genome sequencing (WGS), and, more recently, foundation models, machine learning (ML), and artificial intelligence (AI). These investments aim to accelerate target identification, personalize medicine, measure disease in new ways (e.g., smartwatches), predict outcomes, automate analysis and reporting, improve success rates, and use resources more efficiently.

To realize the value of investments in technology, careful consideration should be given to ensure that the tools available in R&D deliver value.^(p5)^ New data modalities and endpoints, higher volumes of data, novel trial designs, and evolving regulations require innovation in the methods used to collect, analyze, and report evidence for the safety and efficacy of new drugs.^(p6)^ Quantitative scientists also have significant roles in helping to fully realize the value of new technologies in drug development, gaining efficiencies through novel designs and devising new methods to fully assess patient benefit. Open-source software development has emerged as an agile solution for keeping pace with advances in statistical methodology. Recent methodological literature reveals that the standard now includes not only code or data alongside a scientific publication, but also open-source software packages as reference companions.^(p7)^ There has been a conscious decision within pharmaceutical companies to develop open-source software for statistical innovations, with the objective of increasing credibility and facilitating faster adoption in the statistical community.^(p8)^ Here, ‘open-source’ does not only mean access to the original source code, but rather that the software is distributed with an open-source license, explicitly permitting the use of the code according to the license. If code was published without a license, then this would imply that nobody besides the original author could use, copy, distribute, or modify the code,^(p9)^ defeating the purpose of the publication besides transparency.

Here, we examine the open-source statistical software revolution developing in the pharmaceutical industry, the philosophical and technical challenges associated with the adoption of open-source statistical software, and enabling strategies to address its sustainability and rapidly evolving future. We note that the challenges experienced in applied sciences and academia are similar, and many of the solution strategies have parallels in both divisions. Although we discuss academic examples, we keep the focus on statistical analyses in the pharmaceutical industry to manage the scope of our discussion. Due to the foundational importance of open-source licenses, we begin with a definition of them. We then highlight the potential of open-source statistical software development with relevant real-life examples.

## Open-source definition

We aim to adhere to the open-source definition as stated by the Open Source Initiative (OSI), which includes, among others, the following important criteria for open-source licenses^(p10)^: (i) It must be possible to freely redistribute the software (without royalties or other fees); (ii) modified versions of the software (‘derived works’) are allowed to be made and distributed, at least under the same license (the specifics depend then on the particular license choice); (iii) the license must not be specific to a whole software package, that is, parts of the software must be allowed to be used independently and with any other software components when redistributed; and (iv) the license must not restrict the users or the application, for example, it must allow use of the software in a business setting or any research area.

There are corresponding OSI approved open-source licenses, which include popular licenses, such as Apache License (Version 2.0), GNU General Public License (GPL), and the MIT License.^(p11)^ For example, R^(p12)^ is licensed under GPL version 2, and R packages published on the Comprehensive R Archive Network (CRAN)^(p13)^ must be licensed under one of the approved open-source licenses. This ensures that R users can use the base software together with any of the R packages from CRAN in an easy way, without the need to check for license compatibility.

More generally, open-source licenses are central to the success of the open-source ecosystem.^(p14)^ Legal compatibility of software components is a key requirement for any commercial or academic organization and is guaranteed by open-source licenses; specifically important points are that business use is permitted and research areas are not restricted by open-source licenses. The ability to freely combine pieces of code from existing open-source publications is a prerequisite for innovation and development of the software—not only for further open-source software development (which is possible with all open-source licenses), but also for commercial, closed-source software development if the license is ‘permissive’ (e.g., MIT, Apache, and Lesser GPL licenses). The permission for redistribution also allows for sustainable life cycles of open-source software because software maintainers can change over time and inherit and further maintain the work of previous authors.

## Organizational challenges to adopting open source

### The elephant in the room: why should we bother?

The pharmaceutical industry has long relied on closed-source proprietary statistical software as a secure choice. These software types offer documented validation and certified quality, which are included in the licensing royalty fees. However, with rapid innovation in data and methods in the pharmaceutical industry, software requirements are changing, and pharmaceutical companies must identify their system requirements for the future. The product offering of vendors may no longer align with a company's rapidly developing needs. The company then has a choice: work with the vendor to try to make the product fit or develop an open solution for the future.

**Figure 1:**
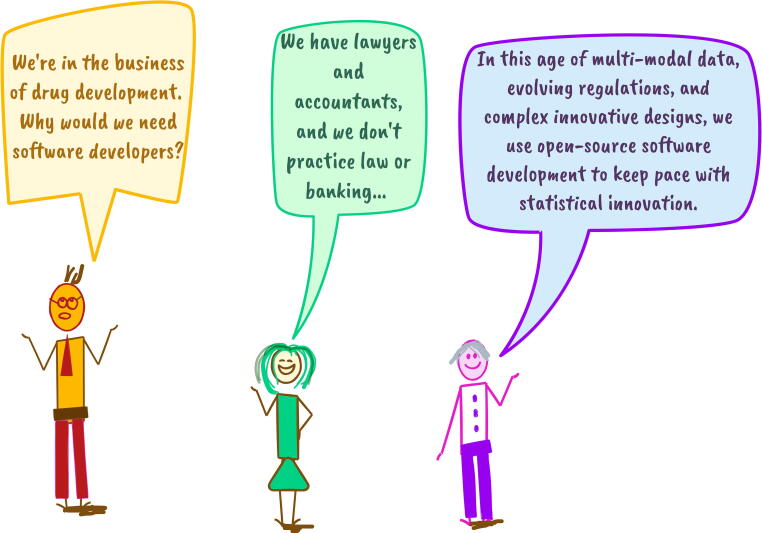
Why do we need statistical software developers?

One of the most fundamental challenges that senior executives pose against developing open-source statistical software in-house is the idea that it is not their core business. As shown in Figure 1, a pharmaceutical executive might wonder: ‘We are in the business of developing medical products to treat patients. So why should we get into internal statistical software development?’ It is a logical question and the response is just as rational, providing a counter example: ‘We also have accountants and lawyers. Although we are not in the business of banking or law, navigating those elements is essential to a successful business’.

In reality, the amount of data in big data has been growing for decades. New digital and imaging measurement modalities generate gigabytes of data per patient. We can integrate data from external sources to inform decisions and, in some cases, supplement clinical trials. We are also implementing more complex clinical trial designs, allowing for adaptation throughout the process, even in confirmatory trials. All of this requires the use of increasingly sophisticated analysis methods. To implement these now and at scale, analytical method development needs to be accompanied by validated software that is reusable, customizable, and flexible enough to handle the nuances of the relevant context. An ad hoc approach to these challenges has its limitations, and we must recognize the need for dedicated Research Software Engineering (RSE) expertise, just as other expertise is valued in other business domains.

Hesitancy to commit to a coding language in an evolving space is another potential hurdle for corporate adoption of open-source software. A current debate revolves around the question of why we should use R, and not Python, or whatever will come next. Python might be a better general programming language and is preferred for ML and AI.^(p15)^ For statistical analyses required for clinical trials, including hybrid data sets (which combine clinical trials with real-world data), R is still advantageous due to the availability of statistical packages and wide adoption in academia, where new statistical methods are often developed. Importantly, large language models are rapidly emerging as a potential tool to simplify the translation of code between programming languages. In addition, we must acknowledge that both R and Python have been established programming languages in data science for more than two decades. It would be a misconception to think that predominant programming languages in a given application field change every few years. As a result, there is potentially greater risk and financial cost associated with remaining stagnant rather than adapting to the advances and forward shift of the field.

### Barriers and silos within the company

Rigid policies and regulation within the pharmaceutical industry are frequently cited as barriers to implementation of innovation in this space.^(p2)^ Variations in the interpretation of regulatory requirements (e.g., the Title 21 CFR Part 11 regulations on electronic records and signatures), can hinder the adoption of open-source statistical analysis software by companies.^(p16)^ These regulations exist to ensure that the data, and the systems in which they are operated on and stored, are of sufficient reliability and quality; the corresponding requirements are defined on a high abstraction level. While health authorities, such as the US Food and Drug Administration (FDA) and European Medicines Agency (EMA), do not mandate specific software, they emphasize the need for thorough documentation of software packages used in submissions, including version and build identification.^(p17)^ Similarly, the ICH E9 guidelines highlight the importance of analysis software validity and reliability for the credibility of numerical results, requiring documentation of testing procedures.^(p18)^

In early 2020, the R Validation Hub, a collaboration to support the adoption of R within a biopharmaceutical regulatory setting, published a white paper introducing ‘A risk-based approach for assessing R package accuracy within a validated infrastructure’.^(p19)^ The framework addresses concerns raised by statisticians, statistical programmers, informatics teams, executive leadership, quality assurance teams, and others within the pharmaceutical industry about the use of R and selected R packages as a primary tool for statistical analysis for regulatory submission work. The overall conclusion was that there is minimal risk in using base R and recommended packages as a component in a validated system for regulatory analysis and reporting.^(p20)^

Beyond regulations, the presence of silos (defined by the Cambridge Dictionary as parts of a company, organization, or system that do not communicate with, understand, or work well with other parts) within companies can also affect the adoption and performance of open-source software.^(p21)^ Silos create inefficiencies in several notable ways: (i) Departmental silos: communication gaps between departments, such as IT and Statistics, hinder the adoption of open-source software. Statistics departments need IT support for infrastructure (i.e., laptops, servers, and cloud-based computing facilities). Lack of communication can lead to frustration and prevent the Statistics department from accessing necessary modern tools; (ii) purpose silos: different systems and programming languages are often used for regulatory submissions versus exploratory statistical analyses. This requires recoding when exploratory analyses become relevant for regulatory use, causing time and resource inefficiencies. The work of the Comparing Analysis Method Implementations in Software (CAMIS) working group, which tries to align the results of different programming languages, shows that this is a nontrivial issue^(p22)^; (iii) project silos: within a company or institution, teams can copy and paste code between projects instead of developing generic, reusable code. This leads to manual adaptation, potential errors, and a lack of resource efficiency, especially as code bases grow more complex over time. Strategic planning for joint development efforts could prevent this.

### Overcoming proprietary mindsets

In the competitive field of pharmaceutical development, where the aforementioned in-house lawyers are protecting intellectual property (IP), organizations might take for granted the need to keep statistical software and code development proprietary. With rather established industry data, reporting, and output standards, companies are well poised to gain efficiencies by using common open-source statistical software and pipelines for standard outputs. For new innovations in statistical analyses, the sharing of code can quickly facilitate adoption,^(p23)^ thereby normalizing new methods for regulators and other stakeholders.

The repeated effort required to keep statistical coding and analysis pipelines proprietary is redundant. Standard statistical analyses are not the subject of innovation, research, or the business itself, but only used as tools to present the evidence generated (e.g., from clinical trials). The IP is the medicine itself. Pharmaceutical companies employ qualified personnel to perform their respective roles, but across companies, the produced data, analysis, and outputs are similar. Efficiency gains could be realized by collaborating and co-developing methods and statistical software. Expediting statistical software availability ensures that the most profitable and beneficial aspects of drug development, the new medical products, can be delivered sooner to patients while being simultaneously more cost efficient.^(p24)^

## Success factors for open-source statistical software

Statistical software is an invaluable tool in quantitative decision making and scientific research. In the top management levels of pharmaceutical companies, emphasis is often placed on the ease of visualizing and interpreting results to inform decision-making, assuming that the analyses and resulting numbers are correct. However, behind the scenes, multiple steps must be taken to ensure the usability and quality of statistical software.

### Usability

Open-source projects often start from code written by an individual for their own use (e.g., to explore a novel statistical methodology), which is eventually published either because of altruistic motives or external pressures and incentives. However, others might sometimes struggle to use the software due to a lack of suitable documentation, intuitive interface, or naming conventions. This results in researchers sometimes preferring to start from the beginning instead of using the already-available software. Simultaneously, the usability of the software has a profound influence on its subsequent success.

Application Program Interface (API) design itself can greatly enhance the usability of software libraries.^(p25)^ Even simple elements, such as the semantics of method signatures (i.e., the names of the methods, the parameters, or inputs and their order) can make a difference. Usability tests can help design efficient and effective user interfaces or client APIs and should be a prioritized part of the development process.

### Quality: accuracy, reliability, and reproducibility by design

The accumulation of scientific knowledge fundamentally relies on the quality and reproducibility of analysis results.^(p26)^^,^^(p27)^^,^^(p28)^ Manual analysis workflows and non-version-controlled code development are major obstacles to achieving reproducibility. To ensure accurate analyses and results, all new software development should incorporate professional workflows and adequate testing. Sanchez *et al.* described key steps for implementing a code quality assurance process that researchers can follow throughout a project to improve their coding practices regarding the quality of the final data, code, analyses, and results.^(p29)^ This includes code style, documentation, version control, data management, testing, and code review. Taschuk and Wilson presented ten simple rules to make research software robust enough to be run reproducibly.^(p30)^ These included version control, documentation, release versioning, build tools, and tests, among others.

To support the development and use of new R packages, risk assessment frameworks and tools have been developed.^(p31)^ For practical purposes, risk assessment criteria can be categorized into package purpose, good maintenance practices, testing coverage, and community usage. The R Validation Hub also created tools such as the riskmetric R Package^(p32)^ and the Risk Assessment application^(p33)^ to simplify risk assessment information gathering. In addition, the Regulatory R Package Repository working group was established and aims to create a repository of R packages that are deemed reliable enough to be used for medical data analysis.^(p34)^ The idea is to help users differentiate easily between high-quality and standard packages and, thus, make the most informed decisions when selecting software for a given purpose. The development of statistical software — from the first prototype to high-quality reliable packages — is indeed a long-term process that can be conceptualized in distinct ‘phases’, analogous to the phases of drug development.^(p35)^ It is essential to help software users determine the development phase a piece of software has reached, enabling them to make informed decisions.

Considering that no method is expected to perform best across all settings, method selection can also be informed by simulation studies tailored to the specific context under consideration. Implementing such simulation-based comparison studies in an unbiased way requires advanced skills and major efforts.^(p36)^ Open-source software that implements such complex studies can, in the long run, contribute to better informed methodological decisions. Recent examples include subgroup identification methodologies^(p37)^ and methods for drawing pertinent causal inferences in trials with intercurrent events.^(p38)^

### Collaboration for efficiency and reliability

For both industry and academia, publication of open-source statistical code is important. External stakeholders of pharmaceutical companies, such as the regulatory authorities, health insurance payer institutions, legislators, and the public, rightfully want to be informed on how the data from clinical trials were analyzed and summarized into the final published results. Here, the possibility of sharing clinical trial data is a first step,^(p39)^ but the availability of the full stack of statistical software used in the data analysis is an important second step.

In academia, the availability of software used to implement new statistical methods in online appendices is an increasingly common requirement for publishing. With the publication of open-source software, the next logical step is for cross-organizational collaboration on this common public code base. In addition, the software becomes more reliable when the burden of developing, testing, and addressing user requests is shared across more developers.

Within the pharmaceutical industry, several initiatives drive the synergistic collaboration between companies on common open-source statistical software: (i) The R Consortium provides support to the R Foundation and to the key organizations developing, maintaining, distributing, and using R software through the identification, development, and implementation of infrastructure projects.^(p40)^ It hosts several working groups that work on specific topics, such as submissions, tabulations, certifications, and repositories; (ii) The openstatsware working group aims to engineer selected R packages to fill gaps in the open-source statistical software landscape and to promote good software engineering practices within biostatistics^(p41)^; (iii) the PHUSE organization is a global community and platform for the discussion and progress of statistical programming topics^(p42)^; and (iv) PSI is a community dedicated to leading and promoting the use of statistics within the healthcare industry.^(p43)^

## Success strategies: case studies and the power of collaborative ecosystems

### Insights from recent case studies

Table 1 provides examples of five open-source software packages developed to implement statistical innovations in response to evolving business needs, as well as increasing the efficiency of established statistical reporting. The first example, rbmi, implements a computationally efficient, fully deterministic approach to imputing missing data in clinical trials with longitudinal endpoints that control type I error and are consistent with the research question.^(p44)^^,^^(p45)^ The second example, crmPack, implements a range of model-based dose escalation designs, from classical to modern continual reassessment methods (CRMs), based on dose-limiting toxicity endpoints and dual-endpoint designs (e.g., plus biomarker or clinical endpoints), and permitting Bayesian and non-Bayesian inference.^(p46)^^,^^(p47)^ The third example, bonsaiforest, aims to more accurately obtain treatment effect estimators for subgroups in the context of binary and time-to-event endpoints.^(p48)^ The fourth example, tern, is a Table, Listings, and Graphs (TLG) library for common outputs used in clinical trials.^(p49)^ It is merely one example of how new software can help to make statistical reporting more efficient, moreover enabling open source-based submissions to regulatory authorities. The final example, rpact, is a fully validated package that enables the design, simulation, and implementation of fixed and adaptive clinical trial designs.^(p50)^ Each of these examples solves relevant analysis challenges in drug development using rigorous, validated methods that have been published in peer-reviewed journals. The fact that the software is publicly available helps to ensure that the community can adopt it quickly, identify deficiencies and errors, contribute ideas and additions, and resolve issues promptly.

**Table 1:** Case studies: open-source packages for common statistical analyses in drug development.

Name	Application	Relevance	Technical information
rbmi	Implements novel deterministic missing data imputation for trials with continuous longitudinal outcomes	Addresses missing data caused by dropout or other intercurrent events in computationally efficient manner more consistent with research objectives compared with alternative methods	Latest version is 1.5.2, published on November 20, 2025, licensed under Apache License version 2 or greater: https://insightsengineering.github.io/rbmi/

crmPack	Implements wide range of model-based dose escalation designs	Flexible package that implements standard and customized designs with dose-limiting toxicity endpoint and dual endpoints (e.g., plus biomarker or clinical endpoints), and permitting Bayesian and non-Bayesian inference	Latest version is 2.0.1, published on December 4, 2025, licensed under GNU General Public License (GPL) version 2 or greater: https://openpharma.github.io/crmPack/

bonsaiforest	Obtains more accurate treatment effect estimators for subgroups in context of binary and time-to-event endpoints	Interpreting subgroup-specific treatment effect estimates requires great care due to the smaller sample size of subgroups and large number of investigated subgroups. This method borrows information across subgroups to appropriately shrink the subgroup estimate toward the population estimate	Latest version is 0.1.1, published on September 27, 2024, licensed under Apache License version 2.0: https://insightsengineering.github.io/bonsaiforest/

tern	Creates common tables, listings, and graphs used in clinical trials	Enables clinical trial analysis in R up to regulatory standards; in particular, provides code for producing clinical trial outputs ready for submission to regulatory authorities	Latest version is 0.9.10, published on December 18, 2025, licensed under Apache License version 2.0: https://insightsengineering.github.io/tern/

rpact	Implements clinical trial planning, design, simulation, evaluation, and analysis	Fully validated free of charge R package with code, vignettes, an app, and training to enable design of fixed and adaptive clinical trials	Latest version is 4.3.0, published on December 16, 2025, licensed under GNU Lesser General Public License (LGPL) version 3.0: https://www.rpact.org

These case studies share a common factor: a collaborative ecosystem for quality and sustainability. Here, we deem a software project sustainable if the resulting software can be used over a prolonged period, thus eventually saving more time with its use compared with the time it took to develop the software overall. Although these case-study software projects were initially developed under the sponsorship of a primary backer, their continued maintenance and evolution are now made possible through the pooled resources of multiple sponsors and the active participation of a wide user community. This collaborative model ensures that the software remains relevant, up-to-date, and able to meet the evolving needs of the pharmaceutical industry. In addition, it fosters a sense of shared ownership and responsibility among the users, who are invested in the success of the software and are more likely to contribute to its ongoing development.

### Building sustainable teams for the future

Analysis methods are increasing in their complexity, and the volume of data in general is growing rapidly. Implementing Research Software Engineering (RSE) as a recognized and dedicated profession, jointly with basic RSE skills education for all statisticians, would provide the special expertise needed to link both the complexity of statistical analysis and burden of data volume.^(p51)^ RSE is the use of software engineering principles in research projects to create reproducible, reusable, reliable, and accurate software. It requires an understanding of both the research subject and software development to ensure software can effectively support research outcomes.^(p52)^

This needs to be based on the honest recognition that software engineering is more than just being able to code in a programming language; in particular, most statisticians do not have software engineering skills. Research software engineers are crucial collaborators with scientists of other professions, and RSE collaborations would include not only multidisciplinary collaboration within an organization, but also structured cooperation across both academia and industry.

Given their expertise in research, statistics, and software engineering, RSEs are highly sought-after by both academia and industry. To attract and retain these valuable talents, organizations must cultivate an environment that recognizes and rewards their contributions. Within academia, this begins with acknowledging software as a valuable research output and incentivizing its development.^(p30)^^,^^(p53)^ Although publications remain a cornerstone of academic recognition, a growing movement advocates for software to be considered a first-class research output, granting due credit to contributors and maintainers. Creating dedicated career paths for RSEs that extend beyond postdoctoral positions, including leadership roles, is needed, similar to those of librarians and lecturers (e.g., as implemented by the Quantitative Biology Center at the University of Tübingen, or the National Coordination Point Research Data Management in The Netherlands^(p54)^).

Likewise, the industry demand for software engineers proficient in data science languages, such as R and Python, is intensifying,^(p55)^ extending beyond statistics to fields such as chemistry.^(p56)^ Competition for these highly sought-after individuals is fierce, with tech companies also vying for their skills. Consequently, the recruitment process can be resource-intensive, making the retention of RSEs through competitive career paths and compensation crucial. It is essential to recognize that RSEs are not merely support staff but integral members of the research ecosystem. Providing them with competitive salaries, seniority levels, and opportunities for growth comparable to those of methodology experts or managers is essential to maintain a motivated and productive workforce.

## Concluding remarks

In the highly regulated pharmaceutical development industry, the move to open-source statistical software is nothing short of revolutionary. At the time of this writing, we are in the middle of this trajectory as many large pharmaceutical companies make this transition. New open-source statistical software packages are emerging regularly in parallel with analytical methods. Registration packages have already been submitted to health authorities [across FDA, EMA, National Medical Products Administration (NMPA), and Pharmaceuticals and Medical Devices Agency (PMDA)], with analyses generated entirely using open-source statistical software.^(p57)^^,^^(p58)^

Multiple factors have brought the industry to this point, including advances in the technology of other disciplines, newer data modalities, and more complex study designs driving the development of new analytical methods. After decades of relying almost exclusively on closed-source, proprietary statistical software, a key goal is to practice statistical software engineering in a sustainable way. This is accomplished by publishing methods and software as open source, integrating new software in an established ecosystem, organizing long-term maintenance, and adhering to good software engineering practices. This requires overcoming certain organizational mindsets and silos (within and across companies) as well as ensuring that the right team members (including those with necessary RSE skills) are hired and retained to develop and maintain software, while also cross-training colleagues to facilitate this work. To obtain the full potential of open-source software development, it is essential that organizations collaborate more effectively when developing software across both industry and academia settings, ultimately leading to an improved and successful product for all. Here the industry can learn from academia, which has relevant experience with RSE groups and roles, as well as cross-institutional cooperation.

Our goal here is not to imply that one must take an either/or decision on proprietary closed-source versus open-source statistical software, but rather we hoped to highlight that Pandora's box has already been opened, and that open-source statistical software can assist industry and academia with large demands to innovate for the future.

## CRediT authorship contribution statement

**Daniel Sabanés Bové:** Writing – review & editing, Writing – original draft, Supervision, Project administration, Conceptualization. **Heidi Seibold:** Writing – review & editing, Writing – original draft, Conceptualization. **Anne-Laure Boulesteix:** Writing – review & editing, Conceptualization. **Juliane Manitz:** Writing – review & editing, Writing – original draft, Conceptualization. **Alessandro Gasparini:** Writing – review & editing, Writing – original draft. **Burak K. Günhan:** Writing – review & editing, Writing – original draft. **Oliver Boix:** Writing – review & editing, Writing – original draft, Conceptualization. **Armin Schüler:** Writing – review & editing, Writing – original draft, Conceptualization. **Sven Fillinger:** Writing – review & editing, Writing – original draft. **Sven Nahnsen:** Writing – original draft. **Anna E. Jacob:** Writing – review & editing, Project administration. **Thomas Jaki:** Writing – review & editing, Writing – original draft, Conceptualization.

## Data Availability

No data was used for the research described in the article.
